# Histological outcomes after focal high-intensity focused ultrasound and cryotherapy

**DOI:** 10.1007/s00345-015-1561-0

**Published:** 2015-05-06

**Authors:** Taimur T. Shah, Veeru Kasivisvanathan, Charles Jameson, Alex Freeman, Mark Emberton, Hashim U. Ahmed

**Affiliations:** Division of Surgery and Interventional Science, Urology Research Group, UCL, Room 4.23, 4th Floor, 132 Hampstead Road, London, NW1 2PS UK; NIHR UCLH/UCL Comprehensive Biomedical Research Centre, London, UK; Department of Urology, UCLH NHS Foundation Trust, London, UK; Department of Urology, Whittington Hospital NHS Trust, London, UK; Department of Histopathology, UCLH NHS Foundation Trust, London, UK

**Keywords:** Focal therapy, Prostate cancer, Minimally invasive therapy, High-intensity focussed ultrasound, HIFU, Cryotherapy, Histology, Pathology, Trial design

## Abstract

**Introduction:**

Focal therapy has increasingly become an accepted treatment option for patients with localised prostate cancer. Most follow-up protocols use a mixture of protocol biopsies or “for cause” biopsies triggered by a rising PSA. In this paper, we discuss the histological outcomes from these biopsies and their use in guiding subsequent management and trial development.

**Methods:**

We conducted a literature search and reviewed the post-treatment biopsy results from studies on focal HIFU and focal cryotherapy. We subsequently reviewed the results of three recently published consensus statements released discussing many of the issues concerning focal therapy.

**Results:**

Research suggests that 1 in 5 of all post-treatment biopsies after focal therapy are positive. However, the majority of these seemed to be from the untreated portion of the gland or met criteria for clinically insignificant disease. The histological outcomes from focal therapy are promising and confirm its effectiveness in the short to medium term. Furthermore re-treatment is possible whilst maintaining a low-side-effect profile.

**Conclusion:**

Debate is ongoing about the clinical significance of various levels of residual disease after focal therapy and the exact threshold at which to call failure within a patient who has had focal therapy.

## Introduction

The emergence of focal therapy as a potential and major shift in the way we manage localised prostate cancer is a highly debated area of research in uro-oncology. Whilst the concept of the index lesion being the primary driver for progressive prostate cancer seems attractive and its ablation seems logical, the wider acceptance of focal therapy, even using such ablative modalities as cryotherapy and high-intensity focused ultrasound (HIFU) seems limited and questioned. This is especially an issue when regulatory approvals are being considered for device approvals or an approval for an additional indication including the focal ablation of a lesion [1, 2]. However, focal therapy has been increasing in popularity, which is highlighted by the increasing number of focal cryotherapy cases registered in the Cryo Online Database (COLD). The number of focal procedures performed per year has increased from 1 in 1999 to 293 in 2007 [3–5].

Currently, there is an array of treatment modalities being evaluated for the focal treatment of localised prostate cancer. Whilst the two in pole position are HIFU and cryotherapy, having been used for focal therapy for a longer period of time, others under investigation include photodynamic and vascular-targeted photodynamic therapy (PDT/VTP) [6, 7], irreversible electroporation [8], radiofrequency ablation [9], magnetic thermotherapy [10], convective thermal water vapour [11], injectable toxins [12] and focal brachytherapy [13]. The aim of all these technologies is to treat the index lesion within the prostate whilst leaving behind healthy non-cancerous tissue or non-significant disease.

One of the significant areas of debate relates to how success or failure of focal therapy is assessed, regardless of which ablative modality is being delivered. The arguments for and against a number of outcome measures follow a common theme in all studies and considerations given for acceptance and approval for focal therapy. Established prostate-specific antigen (PSA) follow-up criteria used for whole-gland treatments such as ASTRO, Phoenix and the Stuttgart criteria, are difficult to apply to a patient after focal therapy as the untreated normal prostatic tissue will continue to secrete PSA. PSA kinetics and PSA nadir may play a role as PSA secretion from the cancerous lesion is larger than healthy prostate tissue, and Ahmed et al. [14] noted an 80 % decrease in PSA at 3 months after focal HIFU. This decrease persisted at 12 months. The more solid endpoints of metastases and death would require over a decade of follow-up due to the long natural history of even clinically significant prostate cancer. Insisting on such outcomes for changes in clinical practice or regulatory approvals would inevitably stifle innovation. Not only would these findings have limited external validity, reported after 10–15 years but would be prohibitively expensive and resource heavy. Other outcome measures, such as histological outcomes, are clearly needed.

In this paper, we aim to first discuss the existing state-of-the-art with respect to histological outcomes after focal therapy and how best to interpret these in clinical practice. The literature is mainly based on the two most widely used modalities of HIFU and cryotherapy. Secondly, we will discuss histological endpoints from a trial design point of view that might be met with wider acceptance or at the very least, form the premise for further debate.

## Histological changes on radical prostatectomy specimens

### HIFU 

In one of the very first proof of concept studies, Van Leenders et al. assessed the histological changes in unilateral HIFU in men who subsequently underwent a radical prostatectomy (RP) 7–12 days later. This study was set up in the early evaluation of HIFU using the ablate-excise study model to demonstrate that ablation can be achieved, whilst the investigators did not set out to fully ablate all tissue. Macroscopically, well-demarcated circular and ellipsoid lesions were seen. Microscopically, cell necrosis was seen within the core of the lesion, however, and not surprisingly, this was incomplete in six out of nine lesions. Haemorrhage with hyperplastic epithelium and reparative changes was also seen at the borders of the lesion [15].

**Fig. 1:**
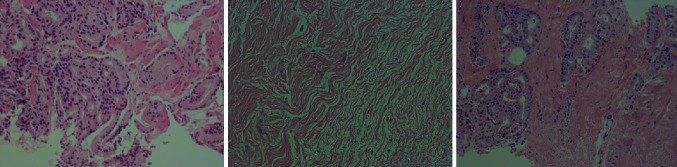
*Left image* shows a Gleason 3 + 4 adenocarcinoma, *middle image* shows typical changes after HIFU with no discernible glands, viable cells or tumour with presence of fibrosis and elastotic collagen, *right image* shows recurrent adenocarcinoma after HIFU

Napoli et al. [16] performed a similar study in five patients with RP performed 7–14 days after HIFU treatment. They also found extensive coagulative necrosis but with no viable tumour within or at the boundaries of the treated lesion. Subsequently, over time, there is development of fibrosis and elastotic collagen (Fig. 1).

Both these studies observed the presence of multi-focal lesions outside of the treated lesion and thus emphasise the need for accurate pre-operative staging and disease localisation.

### Cryotherapy

In 1991 Onik et al. [17] showed complete coagulative necrosis and accurate visualisation of the ice ball under transrectal ultrasound (TRUS) in six dogs treated with cryotherapy. Larson et al. examined exactly the effects of this technique for cryotherapy in five patients who were already scheduled to go undergo salvage prostatectomy 2–3 weeks later. Histologically, squamous metaplasia of glandular epithelium with haemorrhage and a zone of coagulative necrosis and devitalisation was seen spreading from a central core [18]. However, similar to the HIFU studies, incomplete cell death on occasions has also been noted with cryotherapy [19].

### Post-treatment biopsies

Earlier work with protocol-driven TRUS biopsies in 167 out of 176 patients treated with whole-gland cryoablation showed persistent tumour in 38 %. However, in this study, the exact histological data and thus clinical significance of the biopsy results were not mentioned [19]. Similarly, Crouzet et al. [20] performed post-treatment biopsies in 774 patients from cohort of 1002 patients treated with whole-gland HIFU procedures. Overall, 37 % of these biopsies were positive.

Donnelly et al. also performed a randomized control trial (RCT) where 244 men with T2/3 prostate cancer were randomised to either whole-gland cryotherapy or radiotherapy. At a median follow-up of 100 months, they found no difference in disease progression between the two groups. They also found a higher number of positive biopsies at 36 months in the radiotherapy arm (28.9 compared–7.7 % with cryotherapy) [21].

Reviewing the results of eight studies assessing focal cryotherapy, 98/391 (25 %) of all post-treatment biopsies were positive [3, 22–28] (Table 1). No information on grade or location for the 42 positive biopsies from the COLD registry paper by Ward et al. was available. Thus, reviewing the results from the remaining papers shows that 86 % (48/56) of positive biopsies were either from an untreated portion of the prostate or met with criteria of insignificant lesions (Gleason 3, ≤2 cores positive).

Six of these studies mentioned the initial method of diagnosis and only two used transperineal mapping biopsies whilst all the others relied on TRUS biopsies for pre-operative planning. The two studies that did use mapping biopsies had a 13–19 % post-procedure positive biopsy rate [22, 27].

Similarly, from the six studies assessing biopsies after focal HIFU, 22 % (39/175) were positive [14, 29–33] (Table 2). Excluding the four positive biopsies from the paper by Muto et al., as no data on location or histological grade were available, 63 % (22/35) of the positive biopsies were either insignificant or from the untreated part of the gland (Table 2).

**Table 1:** Results from focal cryotherapy studies mentioning post-treatment histological outcomes

Study	Total number of patients	Treatment modality	Biopsy method for initial diagnosis	Protocol or for-cause biopsy method	Number of patients with a positive post-procedural biopsy/total number of biopsied patients	Biopsy significance	Subsequent treatment
Bargawi et al. [65]	62	Cryo	Template mapping	Protocol 12-month TRUS biopsy	12/62 (19 %) 1/12 bilateral 7/12 ipsilateral 4/12 contralateral side	7/8 NS ipsilateral 3/4 NS contra	AS (5) Redo cryo (5) RRP (2)
Bahn et al. [23]	73	Cryo	Doppler TRUS	Protocol and for cause 6- to 12-month TRUS biopsy	12/48 (25 %) 1/12–ipsilateral 11/12 contralateral side	1/1 NS ipsilateral 9/11 NS contralateral	AS (8) Redo cryo (1) brachy (1) ADT (1)
Ward and Jones [3]	1149	Cryo	NA	For cause TRUS biopsy	42/162 (26 %)	NA	NA
Truesdale et al. [24]	77	Cryo	TRUS	For cause TRUS biopsy	10/22 (45 %) 2/10 ipsilateral 7/10 contralateral 1/10 bilateral	NA	NA
Lambert et al. [25]	25	Cryo	TRUS	For cause or PSA nadir <50 % TRUS biopsy	3/7 (43 %) 1/3 ipsilateral 2/3 contralateral	3/3 NS	Redo cryo (3)
Ellis et al. [26]	60	Cryo	NA	Protocol 12-month TRUS biopsy	14/35 (40 %) 1/14 ipsilateral 13/14 contralateral	NA	Redo cryo (12) Lost to FU (1)
Onik et al. [27]	55	Cryo	Template mapping	For cause and protocol 12-month TRUS Biopsy	Protocol 0/26 For cause 4/4 in untreated portion of gland	NA	Redo cryo (4)
Bahn et al. [27]	31	Cryo	Doppler TRUS	Protocol 6-month TRUS biopsy	1/25 (4 %) 1/1 in untreated portion of gland	NA	Redo cryo (4)

*Cryo* cryotherapy, *TRUS* transrectal ultrasound-guided biopsy, *NA* not available, *NS* non-significant, *ADT* androgen deprivation therapy, *AS* active surveillance

**Table 2:** Results from focal HIFU studies mentioning post-treatment histological outcomes

Study	Total number of patients	Treatment modality	Biopsy method for initial diagnosis	Protocol or for-cause biopsy method	Number of patients with a positive post-procedural biopsy/total number of biopsied patients	Biopsy significance	Subsequent treatment
Muto et al. [29]	29	HIFU	TRUS	Protocol 6–12 months TRUS	3/28 at 6 months 4/17 at 12 months	NA	ADT
Fegoun et al. [30]	12	HIFU	NA	Protocol 12 months TRUS	1/12	1/1 NS	Redo HIFU (1)
Ahmed et al. [14]	20	HIFU	Template mapping	Protocol 6 months mpMRI + TRUS targeted of treated side only	2/19 ipsilateral	2/2 NS	AS (1) Redo HIFU (1)
Ahmed et al. [31]	41	HIFU	Template mapping	Protocol 6 months mpMRI + TRUS targeted of treated side only	9/39 ipsilateral	6/9 NS	AS (5) Redo HIFU (4)
Dickinson et al. [32]	88	HIFU	NA	For cause mpMRI + TRUS targeted	20/72 ipsilateral	10/20 NS	NA
Velthoven et al. [33]	27	HIFU	TRUS + concordance with mpMRI lesion 2 months after biopsy	For cause (rising PSA)	3/5 contralateral	3/3 contralateral	Contra-lateral HIFU (3)

*HIFU* high-intensity focused ultrasound, *TRUS* transrectal ultrasound-guided biopsy, *NA* not available, *NS* non-significant, *ADT* androgen deprivation therapy, *AS* active surveillance

However, results such as these have to be interpreted with caution as they are from a heterogeneous group of for-cause, protocol-driven, bilateral or targeted biopsies. It would be expected that for-cause biopsies would be more likely to be positive as they are performed for a clinical suspicion of recurrence. However, studies that only conduct for-cause biopsies, means that those with stable PSAs are selectively not subjected to verification biopsies after treatment. If protocol biopsies are performed then these should be aimed at determining initial treatment success and thus should be targeted at the treated lesion. Additionally, performing biopsies of untreated areas is not assessing treatment success but rather the accuracy of pre-operative assessment.

Eleven studies mentioned the subsequent management of 62 patients in total who had a positive post-treatment biopsy. 61 % (38/62) elected for redo focal therapy and 31 % (19/62) chose active surveillance (AS), whilst 8 % (5/62) had either external beam radiotherapy (EBRT), RP or androgen deprivation therapy (ADT). The ability to retreat with curative intent without significant additional morbidity is a major advantage of focal therapy over whole-gland treatments such as RP or EBRT.

Berge et al. [34] recently published the results of 130 patients undergoing a second HIFU procedure of which 19 underwent a second redo session and one had a third session. Overall, this group was formed from a cohort of 359 patients and thus represented the 36.2 % who needed repeat treatment based on biochemical, histological or imaging (mpMRI) failure. No cancer-specific deaths were reported in this group. Fifty-six men (43 %) did fail a second time. Forty underwent TRUS biopsy and 22 were positive. Their results also showed that side effects were not greatly increased with an increase in pad usage from 2.7 to 9 % (*p* < 0.001) and no effect on potency (*p* = 0.9). Blana et al. [35] had also previously shown a minimal impact on quality of life with a second session of HIFU.

A significant portion chose AS, and there is indirect evidence to suggest that this may be an acceptable option for patients with positive post-treatment biopsies. Data from an AS series of 450 patients diagnosed on TRUS biopsy (and therefore approximately 30 % of whom would have been true intermediate risk) shows a 10-year cancer-specific survival of 97.2 % with only five deaths. A recent update which now included 993 men with up to 16-year follow-up showed a 15-year actuarial cancer-specific survival of 94.3 % [36, 37]. Another indirect source of evidence is studies assessing the outcomes from patients who have post-radiotherapy positive biopsies. 21–32 % of patients may have positive biopsies. The results show that although patients with a positive biopsy have a poorer outcome their 5-year biochemical disease-free survival (bDFS) was still high at 83.3–93.8 % compared to 97.5 % for those with a negative biopsy [38]. In contrast though, Zelefsky assessed 10-year oncological outcomes and found that the group with positive biopsies had only a 3 % PSA relapse-free survival and a 69 % metastases-free survival compared to 59 and 90 %, respectively, for patients with negative biopsies. Zelefsky did have a third group of patients who had positive biopsies showing severe treatment effect. This group did not have a significantly different outcome when compared to those with negative biopsies [39]. Thus, a positive biopsy has to be reviewed carefully before a clinical significance can be implied. Similarly, a positive surgical margin after RP does not always convey a poorer outcome [40].

## Discussion and focal therapy trial design

Knowing this data, the questions that need be asked before designing an appropriate trial are “when to biopsy?”, “How to biopsy?” and “How to interpret the results?”.

It is accepted that a well-designed RCT is high-level evidence upon which treatment decisions can be based. However, designing an appropriate prospective trial to assess the follow-up of patients after focal therapy carries its own challenges. Successfully randomizing men to different surgical treatments in prostate cancer can be challenging.

The initial entry point would be from diagnosis of disease. The methodology for diagnosis is important. Pre-operative TRUS biopsy has been shown to be inaccurate particularly when assessing patients for focal therapy. For instance, 23 % of patients having a template biopsy after a previous TRUS biopsy had upgrading, whilst 60 % were found to have bilateral disease [41]. Systematic transperineal and MRI-guided biopsies have been shown to be equally accurate in detecting cancer [42, 43]. Thus, a similar principle should be applied to post-treatment biopsies. Most still perform systematic TRUS biopsies after treatment that may under-sample the treated area and lead to detection of insignificant disease from untreated tissue.

To improve upon, these ultrasound–MRI fusion techniques have been developed. There seems to be potential benefit of this technique over systematic TRUS biopsies with 67 % more significant disease detected; however, the benefit is less clear when comparing cognitive MRI targeting versus a fusion technique [44–46]. Wysock et al. [45] did not show a significantly increased cancer detection rate between the two techniques, but did find improved accuracy for smaller lesions with fusion biopsies. Whilst Cool et al. [46] showed a 100 % increased accuracy for sampling a clinically significant tumour with fusion targeting versus cognitive. Five millimetres of transperineal mapping biopsies is arguably still the gold standard though, with up to 95 % accuracy when compared to RP specimens [47, 48]. Nonetheless, these require a general anaesthetic and due to the higher number of cores taken have a potentially higher side effect profile.

Subsequently, the clinical significance of disease needs to be reviewed before any treatment is offered. Gleason 6 disease has been shown to rarely if ever lead to metastases and thus death [49–51]. Similarly, metastases are very rare with tumour volume <0.5 cc [52]. Combining this data from results from AS series where few deaths occur, it can be reasoned that low-volume Gleason 6 disease may not benefit from interventional treatment [36]. Thus, the ideal focal therapy candidate is likely to be men with Gleason 7 and above disease or high-volume Gleason 6 cancer.

These same factors play a role following focal therapy. If a patient’s disease has been accurately classified pre-operatively, then follow-up can consist of imaging and biopsies only targeted to the treated area. As previously mentioned, although various criteria exist for follow-up, all effectively conclude that a rising PSA can be used to guide further investigation for recurrence and timing of the post-treatment biopsy. An additional factor to consider is the use of mpMRI to follow up treated lesions and thus triggers a biopsy if residual or recurrent disease is detected.

After focal ablation, there is a residual inflammation, necrosis and eventual fibrosis. Along with some of the data in our own series, Biermann et al. [53] found that detection of grading of cancer in biopsies taken 6 months post-HIFU was possible (unlike with radiotherapy); thus, it seems reasonable that biopsies can be performed at least as early as 6 months.

With respect to mpMRI a consensus meeting found that 77 % of panellists felt that this is a reliable tool for follow-up [54]. From the series of 42 men by Ahmed et al., nine patients were found to have a positive mpMRI, seven of whom had subsequent positive biopsies. Overall, there is some evidence to support the use of mpMRI as a method for follow-up, with it appearing to form an important part of multi-modal follow-up [55–58].

Subsequently, the clinical significance of post-procedural biopsies needs to be considered. There is limited evidence to suggest that low-grade low-volume residual in-treatment field cancer behaves in a similar way to patients with primary disease of the same pathology. Similarly, there is no data on the natural history of secondary low-risk lesions. As mentioned previously, the safest option would be to apply the same principles as for AS and follow up these patients with repeated imaging with biopsies as deemed necessary. From the reviewed cryotherapy and HIFU papers, redo focal treatment and AS appear to be the most common management options selected for patients with positive biopsies.

We subsequently reviewed the results of three recently published consensus statements released discussing many of these issues concerning focal therapy (Table 3) [59–61]. All consensus meetings and expert opinions are considered as level five evidence, and their findings should be considered with this in mind. However, in the absence of high-level evidence, they present important opinions from experts in the field and highlight areas of uncertainty.

**Table 3:** Consensus meeting results

	Inclusion	Exclusion	Pre-op biopsy strategy	Biopsy end point	PSA	MRI	Treatment failure	Re-treatment
Van den Bos et al. [61] Multi-stage Delphi process	3 + 3 with “substantial cancer” 3 + 4 >10-year life expectancy PSA > 15 with caution	Clinically insignificant disease (volume < 0.5 cc)	MRI targeted or fusion with systematic	Negative 12-month biopsies Targeted and systematic	3 monthly but not sufficient as an end point	Alterations in MRI not sufficient as end point	Any cancer in-field of treatment Low-grade, low-volume (Gleason 6, <3 mm) out of field is not considered failure	Acceptable on one occasion
Donaldson et al. [59] RAND/UCLA appropriateness methodology	NCCN intermediate risk disease 3 + 3 with >3–5 mm MCCL >10-year life expectancy Multifocal disease included (secondary lesion of Gleason 6, ≤5 mm can be left untreated)	<5-year life expectancy and those <40 and >80 years with caution WHO performance status 3–4	MRI targeted or template mapping (if no MRI available)	12-month biopsy Targeted only Uncertainty about systematic sampling	Rising PSA may trigger biopsy	Suspicious MRI may trigger biopsy	Cancer in field of equivalent or higher than pre-operative grade Low-grade, low-volume (Gleason 6, <3 mm) in field is not considered failure	≤20 % retreatment rates
Muller et al. [60] Delphi process				12 month biopsy Targeted and systematic	3 monthly No consensus on role of PSA	1st MRI 6 months post-treatment Suspicious MRI should lead to biopsy Further biopsies after 12 months only if suspicious MRI		

Donaldson et al. and Van den Bos et al. separately discussed inclusion and exclusion criteria and both consensus groups agreed that high-volume Gleason 6 (>5 mm MCCL) and Gleason 7 disease are the optimal candidates. They discussed the importance of accurate pre-operative assessment using MRI-targeted or fusion biopsy methods prior to offering a patient treatment. They recommend protocol biopsies at 12 months post-treatment and that a rising PSA or suspicious MRI should also trigger a biopsy. The ultimate end point was a negative 12-month biopsy. However, there was differing opinion on whether these should only be targeted or also systematic or whether residual low-grade and low-volume cancer is considered treatment failure. Donaldson et al. mentioned that biopsies should be targeted rather than systematic in order to reduce sampling of untreated tissue, whilst Muller et al. (another consensus group) commented that systematic biopsies are useful for surveillance of the untreated gland. Re-treatment was considered acceptable and Donaldson et al. commented that overall re-treatment rate should be below 20 % since it was argued that retreatment was a positive attribute of the strategy.

The final point to consider when designing such a trial is the comparator arm. The two most common radical whole-gland treatments are radiotherapy or RP. Neither has been assessed head-to-head in a RCT. Retrospective reviews have shown surgery to have better oncological outcomes; however, even with the best matching, bias can never be fully excluded [62, 63].

The role of focal therapy will ultimately help decide the most appropriate comparator arm. We feel that the current role of focal therapy is similar to that of tissue preservation in almost all other solid organ cancers (bar ovarian), in that, it leads to a minimal decrease in quality of life for the patient whilst providing acceptable cancer control. At least in the short to medium term, the data suggest that focal therapy may be meeting these aims. However, few studies have long-term follow-up and a systematic review showed a varying bDFS of between 86.2 % at 8 years and 60 % at 5 years [64].

Thus, in the first instance, patients with localised intermediate- to high-risk disease should be compared to those undergoing radical treatment with either EBRT or RRP. The aim of this study would be to show non-inferiority for short- to medium-term oncological outcomes with a superior side effects profile. Repeat treatments are possible with focal therapy, and a second treatment would not be considered a treatment failure unless there was a significant reduction in the two outcome measures mentioned.

## Conclusion

Research suggests that one in five of all post-treatment biopsies after focal therapy is positive. However, the majority of these seemed to be from the untreated portion of the gland or met criteria for clinically insignificant disease. The histological outcomes from focal therapy are promising and confirm its effectiveness in the short to medium term. Furthermore, re-treatment is possible whilst maintaining a low side effect profile. Debate is ongoing about the clinical significance of various levels of residual disease after focal therapy and the exact threshold at which to call failure within a patient who has had focal therapy.
